# Genome-Wide Association Studies of Seed Performance Traits in Response to Heat Stress in *Medicago truncatula* Uncover *MIEL1* as a Regulator of Seed Germination Plasticity

**DOI:** 10.3389/fpls.2021.673072

**Published:** 2021-06-04

**Authors:** Zhijuan Chen, Joseph Ly Vu, Benoit Ly Vu, Julia Buitink, Olivier Leprince, Jerome Verdier

**Affiliations:** Institut Agro, Univ Angers, INRAE, IRHS, SFR 4207 QuaSaV, Angers, France

**Keywords:** GWAS, *Medicago truncatula*, heat stress, seed germination, plasticity

## Abstract

Legume seeds are an important source of proteins, minerals, and vitamins for human and animal diets and represent a keystone for food security. With climate change and global warming, the production of grain legumes faces new challenges concerning seed vigor traits that allow the fast and homogenous establishment of the crop in a wide range of environments. These seed performance traits are regulated during seed maturation and are under the strong influence of the maternal environment. In this study, we used 200 natural *Medicago truncatula* accessions, a model species of legumes grown in optimal conditions and under moderate heat stress (26°C) during seed development and maturation. This moderate stress applied at flowering onwards impacted seed weight and germination capacity. Genome-wide association studies (GWAS) were performed to identify putative loci or genes involved in regulating seed traits and their plasticity in response to heat stress. We identified numerous significant quantitative trait nucleotides and potential candidate genes involved in regulating these traits under heat stress by using post-GWAS analyses combined with transcriptomic data. Out of them, *MtMIEL1*, a RING-type zinc finger family gene, was shown to be highly associated with germination speed in heat-stressed seeds. In *Medicago*, we highlighted that *MtMIEL1* was transcriptionally regulated in heat-stressed seed production and that its expression profile was associated with germination speed in different *Medicago* accessions. Finally, a loss-of-function analysis of the *Arabidopsis MIEL1* ortholog revealed its role as a regulator of germination plasticity of seeds in response to heat stress.

## Introduction

Legume is an economically important crop family, that includes many plant species such as soybean, pea, common bean, and chickpea. *Medicago truncatula* is a model plant of legumes originating from the Mediterranean region (Barker et al., 1990), which has been intensively studied for legume research. Grain legumes provide abundant proteins, minerals, and other nutrients for human and animal diets and play a vital role in global food security. However, climate change threatens crop production by causing reduced yield and loss of product quality. In the context of global warming, legume seed production suffers from environmental stresses, including heat stress, and legume crops need to be improved toward higher phenotypic plasticity (Vadez et al., 2012; Scheelbeek et al., 2018). Indeed, while the local adaptation of a genotype is genetically determined under certain environmental conditions (Tognetti et al., 2019), phenotypic plasticity can generate different phenotypes according to the environment (Valladares et al., 2006). This variation is created by the interplay of genetic and environmental factors. Understanding the genetic basis of local adaptation and phenotype plasticity is highly relevant in our current climate change context. Heat stress affects the proper development of female and male gametophytes, leading to impaired double fertilization and decreased seed number (reviewed in Liu et al., 2019). Also, heat stress during early embryogenesis was shown to reduce grain yield in soybean and mungbean (Siebers et al., 2015; Patriyawaty et al., 2018). During seed development, maturation was shown to affect seed vigor. Seed vigor is a composite term that includes homogeneous and rapid germination and seedling establishment under a range of contrasted environmental (i.e., stress) conditions (Finch-Savage and Bassel, 2016). In *M. truncatula* (Verdier et al., 2013; Righetti et al., 2015), the different vigor traits are acquired sequentially, from seed filling until the late phase of seed maturation (reviewed in Leprince et al., 2017). So far, genetic determinants of seed vigor in *Medicago* have been explored, mostly by QTL identification using several populations of recombinant inbred lines resulting from crosses between contrasting accessions (Vandecasteele et al., 2011). These studies led to identifying several key regulatory genes of the late maturation phase, such as *MtABI5* (Zinsmeister et al., 2016) and *MtHSFA9* (Zinsmeister et al., 2020). In *Medicago* (Righetti et al., 2015), like many other species (Finch-Savage and Bassel, 2016; Penfield and MacGregor, 2017), seed vigor is also drastically affected by environmental conditions during seed development. This highly plastic response from the offspring to the environment is considered a bet-hedging strategy to ensure the dissemination of the species. In this respect, one of the most studied germination vigor traits is dormancy (for review, Penfield and MacGregor, 2017). In legume seeds, such as *M. truncatula* seeds, we distinguish two types of dormancy, which are physical and physiological dormancies (according to the definition of Baskin and Baskin, 2004). Physical dormancy is mainly controlled by the seed coat permeability, which prevents seed imbibition. However, physiological dormancy is regulated by the embryo and endosperm molecular signals via the ratio of abscisic acid content (ABA), acting as germination repressor and gibberellic acid (GA), allowing germination. For example, in *M. truncatula*, a slight increase in the seed coat properties regulating seed imbibition and physical dormancy was observed when plants were grown in 35°C/15°C compared with 25°C/15°C conditions (Renzi et al., 2020). While widespread germination via a decrease in germination speed or a delay of germination until favorable conditions are advantageous for wild species dissemination, it is not a desirable trait for crops. Furthermore, the plastic response of the germination of seeds produced under environmental conditions is also dependent on complex GxE interactions of the regulation of physiological dormancy involving zygotic and maternal tissues (Penfield and MacGregor, 2017; Awan et al., 2018; Geshnizjani et al., 2019; Chen et al., 2020; Renzi et al., 2020), and the dynamic balance between ABA and GA is poorly understood and likely to be species-dependent (Penfield and MacGregor, 2017; Chen et al., 2020).

In recent years, a genome-wide association study (GWAS) has been widely performed for the association mapping between genetics and agronomic traits to identify causal loci using populations of natural accessions. Many new statistical models to compute the association mapping have been developed from initially single-locus analyses to recent multi-locus analyses, including the fixed and random model circulating probability unification (FarmCPU) (Liu et al., 2016), which improved the statistic power to control false positives and reduce computing time (for review Tibbs Cortes et al., 2021). In *Medicago truncatula*, a haplotype map (HapMap) population was selected based on their geographical origins and genomic diversity and resequenced using next-generation sequencing technologies to identify single nucleotide polymorphisms (SNP) (Stanton-Geddes et al., 2013). The *Medicago* HapMap population, finally, comprises 226 natural accessions characterized by 4.8 million SNP. This collection has been used to study different aspects of *Medicago* biology, such as different abiotic stresses on vegetative part with salt stress (Kang et al., 2019) and drought stress (Kang et al., 2015), but also more specifically to seeds with seed nutritional content (Chen et al., 2021a) and physical seed dormancy (Renzi et al., 2020).

In this study, we used the *Medicago* HapMap collection to identify putative causal genes/loci associated with the plasticity of germination performance traits of seeds produced under heat stress conditions. We performed genome-wide association studies of seed weight and seed germination speed and homogeneity using 200 accessions from the *M. truncatula* HapMap collection via the FarmCPU algorithm. Post-GWAS analyses and RNA-seq data were used to refine our candidate gene lists related to different seed traits. A candidate gene, *MtMIEL1*, involved in the germination plasticity of seeds produced under heat stress was identified in *M. truncatula* and functionally validated in *A. thaliana*.

## Materials and Methods

### *Medicago* Population and Plant Growth Conditions

From the *M. truncatula* HapMap project (http://www.medicagohapmap.org/hapmap/germplasm), 200 accessions were selected and sown in 3-L plastic pots containing Klasmann–Deilmann substrate number 5. Plants (stage 3 trifolioles) were first vernalized at 8°C for 2 weeks. Then, six replicates of each accession were grown using a dripping watering system with water supplemented with 15/10/30 NPK to assure watering and fertilization homogeneity in the greenhouse, where light intensity (600 W/m^2^), photoperiod (16 h day), hygrometry (50–60% relative humidity) and minimal temperature were controlled. All plants were first produced under optimal conditions at 20°C/18°C day/night with 16 h light photoperiod until the flowering stage described in Vandecasteele et al. (2011). After the apparition of five flowers, individual plants were moved to a neighboring greenhouse chamber with the same growing conditions, except the temperature was set to 26°C/24°C day/night. A real-time recording of growth conditions allowed us to precisely track observed average temperature and humidity conditions during seed maturation, which were 18.9°C (±2.4°C) and 42.9% RH (±9.6%) in optimal conditions and 25.7°C (±2.6°C) and 40.5% RH (±10.5%) in heat stress conditions. The average flowering time for the 200 accessions was February the 16th, with a standard deviation of ±8 days across the HapMap population. Finally, triplicates of plants for each accession were maintained under optimal conditions, and triplicates were grown under heat stress conditions with 26°C/24°C day/night temperature. Mature seeds from 199 accessions were daily collected at pod abscission from both conditions and further dried at 20°C in 44% relative humidity (RH). Seeds were stored hermetically at room temperature before use.

### Phenotyping Seed Traits

The individual seed weights of 199 HapMap accessions were calculated from the average total seed weight per plant. The number of mature seeds per plant was counted using a seed counter (Pfeuffer model Contador), and whole seed weights were determined using a precision balance. The average individual seed weight was calculated for each accession and replicate by dividing total seed weight per plant by the seed number per plant, providing an accurate estimate of the individual seed weight. Before any germination experiments, seeds were first scarified to avoid artifacts due to physical dormancy. Logistics and greenhouse conditions obliged us to optimize the number of accessions to assess germination traits. One hundred twelve *Medicago* HapMap accessions were used to assay germination. Triplicates of 50 seeds were imbibed in 5 ml of water in a 5-cm Petri dish containing one Whatman No1 filter paper at 15°C in the dark. Germinated seeds and speed of germination were monitored automatically for control seed lot using the phenotyping platform PHENOTIC (SFR QUASAV, Angers) (Benoit et al., 2014) and manually for the stressed-seed lot by counting germinated seeds (i.e., protruding radicles >1 mm) every 4 h. Germination speed was calculated from the sigmoidal regression of each accession as the averaged time to reach 50% germination (T50). Germination homogeneity was calculated as the time difference between 80% (T80) and 20% (T20) germination (i.e., T80–T20). Finally, the phenotypic plasticity index of all seed traits was calculated based on the following formula: PLAS = (T_St – T_Ct)/T_Ct, where T_St is the mean value of the trait under heat stress conditions and T_Ct the mean trait value under control conditions.

### Correlation Analysis

Correlations between traits were analyzed using the “rcorr” function of the “Hmisc” package (v4.4-0, Harrell, 2020) in R. A global correlation matrix was performed using the Pearson correlation coefficient, and we selected a *p*-value threshold of 0.05 for statistical significance.

### Normalization of Phenotypic Data

To carry out the genome-wide association studies, we checked and transformed, when necessary, our phenotypic data to reach distribution normality. The Shapiro–Wilk test was performed to test the distribution states of all phenotypic traits. Phenotypic data were transformed to normal distributions using the Box–Cox power transformation procedure (Box and Cox, 1964) using adapted lambda values calculated for each trait. The Shapiro–Wilk tests and the Box–Cox transformations were carried out using the “MASS” package (Venables and Ripley, 2002) available in R.

### Genome-Wide Association Analysis

Identification of single nucleotide polymorphisms (SNP) was obtained by whole-genome sequencing of the *Medicago* HapMap accessions selected in the *M. truncatula* HapMap project (Stanton-Geddes et al., 2013). Using the *Medicago* genome version 5 (Mtv5, Pecrix et al., 2018), more than 4.8 million SNP locations were identified and genotyped in the HapMap accessions. This 4.8 million SNP genotypic dataset was used combined with the HapMap population structure (described in Bonhomme et al., 2014) and the normalized phenotypic dataset regarding seed performances. In addition, the multi-locus model FarmCPU (Fixed and random model Circulating Probability Unification, Liu et al., 2016) was used to perform association analyses described in Chen et al. (2021a) with a *p*-value threshold set to 1%. The quantile–quantile (QQ) and Manhattan plots were generated by the FarmCPU package available in R.

### Post-GWAS Analyses

The PLINK algorithm (Purcell et al., 2007) was used to identify correlated SNP and to correct for the linkage disequilibrium (LD) using the “clump” function. The following options of PLINK were used: “clump-kb 30” and “clump-r2 0.7,” which represent the range of analyzed genomic region (± 30 kb) and the R-squared threshold (0.7) to identify correlated SNP. In addition, enrichment analyses of Mapman functional classes of putative causal genes related to different seed performance traits were performed using the Clusterprofiler package (Yu et al., 2012) using a hypergeometric test with Benjamini–Hochberg correction (*q*-values) and available in R. Mapman functional classes were obtained from *Medicago* annotated proteins using Mercator v.4 (Schwacke et al., 2019).

### Transcriptomic Data

The expression data of *M. truncatula* during seed development under optimal and heat stress conditions were obtained from Chen et al. (2021b), and raw data were stored on NCBI Gene Expression Omnibus (GEO; https://www.ncbi.nlm.nih.gov/geo/query/acc.cgi?acc=GSE160725). The differentially expressed genes (DEG) between optimal and heat stress conditions at the different seed developmental stages were identified using ImpulseDE2 (Fischer et al., 2018) for embryo and endosperm and DEseq2 (Love et al., 2014) for seed coat. DEG threshold was set as adjusted *p*-values below 5% following the Benjamini–Hochberg procedure to control the false discovery rate (FDR) described in Chen et al. (2021b).

### RNA Extraction and qRT-PCR

Total RNAs were extracted from two replicates of about 30 dry mature seeds, 24 h-imbibed and 48 h-imbibed (10°C) seeds of *Medicago* reference genotype A17 that were produced in optimal (20°C/18°C, 16-h photoperiod) and heat stress (26°C/24°C, 16-h photoperiod) conditions. Simultaneously RNA extractions were also performed on dry, mature seeds in triplicates of four natural *Medicago* HapMap accessions (i.e., HM170, HM185, HM279, and HM314) produced under heat stress condition (26°C/24°C, 16-h photoperiod). HapMap genotypes were chosen based on their germination speed, with two belonging to the slowest germination set and two belonging to the fastest germination set. All RNA extractions were performed using Macherey-Nagel NucleoSpin^®^ RNA Plant and Fungi kit following the Alfalfa seeds protocol described in the manufacturers' instructions. Total RNA was quantified using a Nanodrop spectrophotometer ND-1000 (NanoDrop Technologies), then treated with RNase-free DNase I (Thermo Fisher Scientific Inc.). Reverse transcriptions were performed using the iScript^TM^ RT Supermix (Bio-Rad Laboratories, Inc.) from 1 μg of DNAse-treated RNA. cDNA was quantified with SsoAdvanced^TM^ Universal SYBR^®^ Green Supermix (Bio-Rad Laboratories, Inc.) using a CFX96 Touch quantitative real-time PCR (qRT-PCR) Detection System (Bio-Rad Laboratories). The primers that were used for qRT-PCR are provided in Supplementary Table 6. *MtMIEL1* primers were designed on the Primer 3 website (https://bioinfo.ut.ee/primer3/). *MtTCTP* was used as reference gene (Verdier et al., 2008; Zinsmeister et al., 2020). The relative expression levels were normalized according to the 2^−ΔCt^ method.

### *Arabidopsis* T-DNA Insertional Mutants and Seed Germination Assays

The T-DNA insertional *miel1* mutant (Salk_041369) from a Columbia-0 (Col0) background were obtained from the NASC germplasm collection. The primers used for isolation of T-DNA homozygote mutants were generated from the T-DNA Primer Design website (http://signal.salk.edu/tdnaprimers.2.html) and are provided in Supplementary Table 6. *Arabidopsis thaliana* plants (Col0 and *miel* mutants) were grown under standard conditions (20°C/18°C, 16-h photoperiod) in a growth chamber Aralab model fitoclima 600 (Tempcontrol, France). At the flowering time (i.e., after bolting as soon as inflorescence appeared), half of the plants were kept at control condition (20°C/18°C, 16-h photoperiod). Then, half were individually moved to another identical growth chamber (Aralab model Fitoclima 600) under heat stress conditions as described in Malabarba et al. (2021) (28°C/26°C, 16-h photoperiod). Mature seeds produced in both conditions were harvested and dried for 3 days at 44% relative humidity and 20°C. The dry seeds were stored at −20°C before the germination test. Three biological replicates of about 100 seeds obtained from three independent *miel1* and wild-type (Col0) plants were used for germination assays. Freshly harvested seeds were imbibed in 1 ml water in 3-cm Petri dishes containing Whatman No 1 filter paper at 20°C with a 16-h photoperiod. To release dormancy, freshly harvested seeds were stratified at 4°C for 72 h in the dark then transferred to 20°C with 16-h photoperiod for germination.

## Results

### Assessing Seed Performances in Response to Heat Stress in *Medicago* HapMap Collection

**Figure 1:**
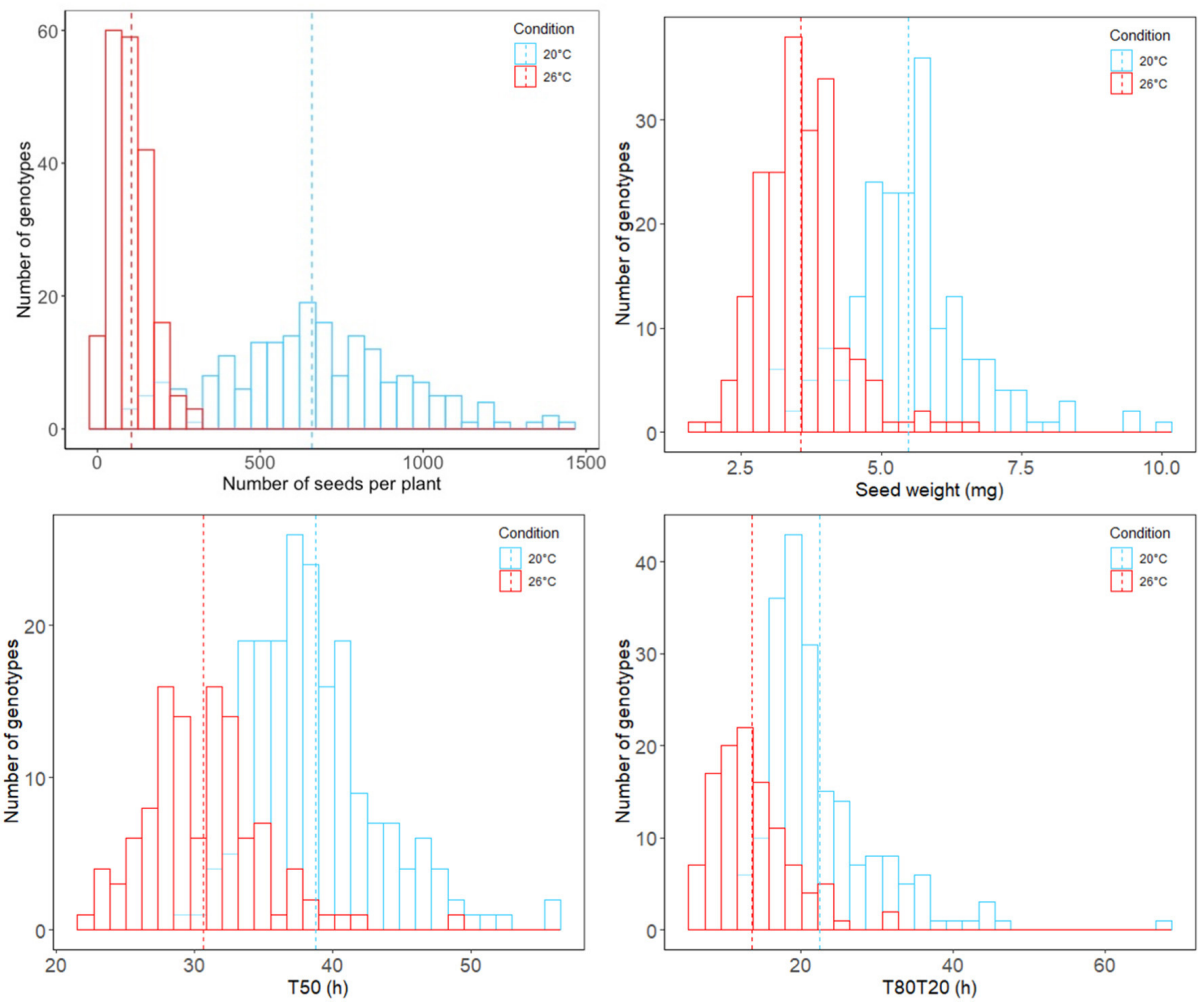
Distribution histograms of analyzed phenotypic data regarding seed traits across the *M. truncatula* HapMap accessions and grown under optimal (blue) and heat stress (red) conditions. Average values across the entire HapMap population are represented in dotted lines.

To evaluate the impact of heat stress on seed yield and vigor, 200 *M. truncatula* accessions from the HapMap collection were grown in triplicate in optimal (20°C/18°C) and supra-optimal temperature (i.e., 26°C/24°C) conditions by applying constant but moderate heat stress from flowering until pod abscission. The intensity of heat stress (26°C/24°C) was decided based on previous experiments performed on *M. truncatula* reference genotype A17 (Righetti et al., 2015) allowing the production of mature seeds but impacting the seed maturation duration. After harvest and moisture content equilibration at 44% RH, we observed a significant decrease in seed yield from plants grown in the heat stress conditions. Across the 200 *Medicago* accessions, the average pod and seed numbers per plant in optimal conditions were 117 and 658, respectively, in contrast to 20 pods and 104 seeds on average from plants grown under heat stress. In addition, an overall 35% decrease in seed weight was observed in accessions produced in heat stress conditions (Figure 1, phenotypes named WEIGHT_C for optimal and WEIGHT_H for heat stress conditions).

The limited seed number produced under heat stress conditions for some accessions directly impacted the number of accessions available for phenotyping seed performance. For instance, the HM059 accession did not produce enough seeds and was discarded. We phenotypically characterized seed germination of mature seeds produced 112 accessions. We observed that about 100% of seeds germinated 6 days after imbibition, no matter if they were produced in optimal or stress conditions. To assess the impact of heat stress on seed vigor, we extracted two germination characteristics: the germination speed (T50, corresponding to the time to reach 50% of germination) and the germination homogeneity (T80T20, duration between 80 and 20% of germination). The heat stress positively impacted germination speed and homogeneity across the population during seed production. Indeed, seeds produced in heat stress conditions displayed an overall tendency to germinate faster and more homogeneously than those produced in optimal conditions (Figure 1, phenotypes named T50_C and T80T20_C for optimal and T50_H and T80T20_H for heat stress conditions).

Even if the overall tendency from all different accessions displayed an increase in seed germination performances and a decrease in seed weight, it is noteworthy that the individual tendency of each accession is more contrasted with some that did not follow the overall tendency. This reflected high phenotypic plasticity within the HapMap population regarding these traits (Supplementary Figure 1 and Supplementary Table 1). Therefore, to assess the phenotypic plasticity (PL) of each accession, we calculated the plasticity index of seed traits obtained in the two contrasted seed production conditions. These plasticity indexes reflected the ability of each accession to produce different phenotypes according to the maternal environment (Supplementary Table 1, phenotypes named WEIGHT_PL, T50_PL, and T80T20_PL).

To determine if seed performance traits measured in different growth conditions were correlated, we performed correlation analyses among them using Pearson coefficient correlation (Table 1). First, we observed a strong positive correlation (0.79) between the weight of seeds produced in optimal and heat stress conditions, suggesting that seed weight is genetically determined in HapMap accessions by the same set of genes in both conditions. Moreover, we observed a weak positive correlation (0.2) between seed weight and speed of germination for seeds produced under control conditions. The correlation was also found for seeds produced under heat stress but was much stronger (0.48). Many studies have documented that seed size is correlated with germination performance, with larger seeds exhibiting better seedling survival rate due to more seed reserve accumulated during seed filling to supply embryo with sufficient energy during germination (reviewed in Finch-Savage and Bassel, 2016). However, the plasticity response of both traits was not correlated, suggesting that there exist different processes regulating seed filling and acquisition of germination performance in response to heat stress. Finally, we observed positive correlations between germination phenotypes measured during heat stress and plasticity indexes, which suggested that mechanisms controlling the germination of seeds produced under heat stress could be similar to those controlling their plasticity. Surprisingly, no significant correlations were identified between the geographical origins of different plant accessions and seed germination traits.

**Table 1:** Correlation matrix between all *Medicago* seed traits and climatic data.

	**TRAITS**	**WEIGHT_C**	**WEIGHT_H**	**WEIGHT_PL**	**T50_C**	**T50_H**	**T50_PL**	**T80T20_C**	**T80T20_H**	**T80T20_PL**
Seed weight	WEIGHT_C	lg								
	WEIGHT_H	0.79								
	WEIGHT_PL	−0.35	0.29							
Seed germination speed	T50_C	0.20	0.16	−0.03						
	T50_H	0.41	0.48	0.11	0.16					
	T50_PL	0.12	0.19	0.09	−0.52	0.75				
Seed germination homogeneity	T80T20_C	0.09	0.14	0.10	0.69	0.18	−0.29			
	T80T20_H	0.17	0.26	0.17	−0.01	0.83	0.75	0.04		
	T80T20_PL	0.02	0.12	0.16	−0.34	0.64	0.79	−0.47	0.82	
Geographical location	Longitude	−0.06	−0.04	0.05	−0.11	0.07	0.14	−0.11	0.07	0.11
	Latitude	−0.03	−0.09	−0.10	−0.05	0.03	0.05	−0.08	−0.04	−0.04
	Altitude	−0.20	−0.27	−0.14	−0.01	−0.13	0.01	0.06	−0.04	0.04

*Pearson correlation coefficients above 0.2 with a p-value below 5% are indicated in red color. Pearson correlation coefficients above −0.2 with a p-value below 5% are indicated in green color. Longitude is indicated by degrees from west to east with negative and positive values, respectively. Latitude is also indicated by degrees from south to north with negative and positive values, respectively*.

### Genome-Wide Association Analyses of Different Seed Traits in Response to Optimal and Heat Stress Conditions and Identification of Putative Causal Genes

**Figure 2:**
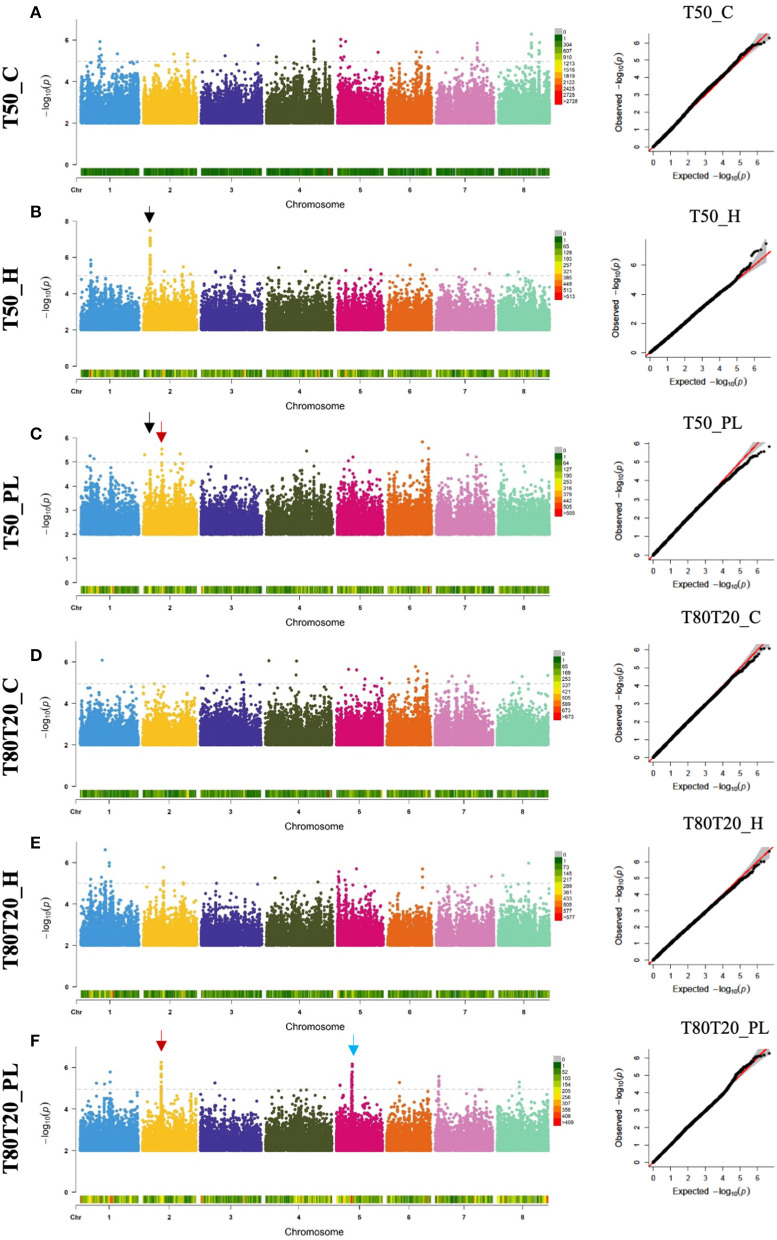
Manhattan plots and the corresponding Q-Q plots from GWAS results regarding seed germination speed (T50) **(A–C)** and germination homogeneity (T80T20) **(D–F)**. Black arrows indicate the common QTN associated with both T50_H and T50_PL corresponding to the *MtrunA17_Chr2g0286331* gene. Red arrows indicate the common QTN associated with both T50_PL and T80T20_PL corresponding to the *MtrunA17_Chr2g0300261* gene. The Blue arrow indicates the highly significant QTNs located in chromosome 5. GWAS, genome-wide association analyses; QTN, quantitative trait nucleotides.

Following phenotypic characterization of HapMap accessions, we used the Box–Cox procedure (Box and Cox, 1964) to transform our phenotypic data that did not display normal distributions. Appropriate lambda values were estimated and used to normalize our phenotypic data to validate the assumption of normality required to perform genome-wide association analyses. After this normalization step, the Shapiro–Wilk test was performed for each phenotype to verify that our phenotypic data reached the normal distribution (Supplementary Figure 2). All lambda values, Shapiro–Wilk *p*-values, and normalized phenotypic data are available in Supplementary Table 1. However, one phenotypic trait did not pass the Shapiro–Wilk test (i.e., WEIGHT_C). Still, it was conserved in subsequent analyses as it displayed acceptable fit to normal distribution based on its distribution histogram and their bell curve (Supplementary Figure 2). Finally, genome-wide association studies were performed on the nine transformed seed phenotypic data using the Fixed and random model Circulating Probability Unification algorithm (FarmCPU, Liu et al., 2016) combined with the *Medicago* HapMap population structure as covariable and the *Medicago* HapMap SNP genotypic dataset (described in Bonhomme et al., 2014 and available at http://www.medicagoHapMap.org). We identified sets of single nucleotide polymorphisms, called quantitative trait nucleotides (QTNs), statistically associated with the different seed traits from these association studies. Manhattan and QQ (quantile-quantile) plots related to seed germination performances are provided in Figure 2, and those related to seed weight are provided in Supplementary Figure 3. To facilitate visualization of these results, we generated “gwas” files that contain all the statistical results of all the SNPs concerning different seed traits, which allow visualization of significant QTNs on genome viewers such as Integrative Genome Viewer (IGV, Thorvaldsdóttir et al., 2013) or JBrowse (Skinner et al., 2009) (provided as Supplementary Tables 2–4).

From these GWAS, we identified highly significant QTNs (*p*-values below 10^−7^) associated with seed weight obtained from optimal (9 QTNs) and heat stress (20 QTNs) growing conditions, as well as 2 QTNs potentially involved in plasticity (Supplementary Figure 3 and Supplementary Table 5). Among these QTNs, one of them, MtrunA17Chr8_49244112, was identified to correlate with seed weight from optimal (WEIGHT_C) and heat stress (WEIGHT_H) conditions. Similarly, highly significant QTNs (*p*-values below 10^−7^) were identified for seed germination traits: 2 and 1 QTNs regarding germination speed of seeds from control and heat stress conditions, respectively, and 3 and 1 QTNs regarding germination homogeneity of seeds from control and heat stress conditions. Moreover, 2 QTNs were identified for plasticity of germination speed. We also observed common QTNs between germination traits located on chromosome 2: MtrunA17Chr2_6710478 common between T50_H and T50_PL and MtrunA17Chr2_18061650 common between T50_PL and T80T20_PL (Figure 2).

**Figure 3:**
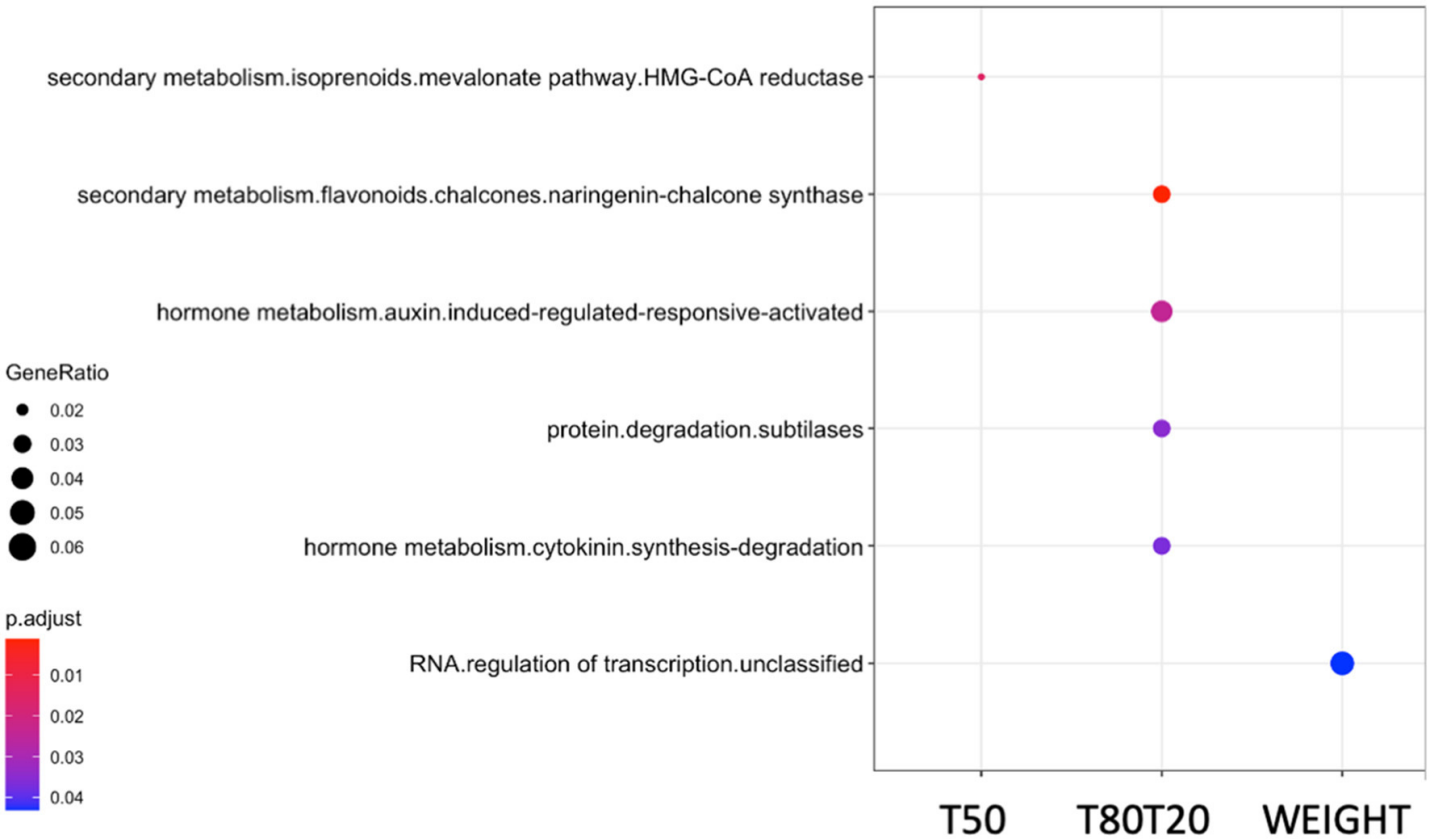
GSEA analysis of candidate gene lists obtained from GWAS with different seed traits: germination speed (T50), germination homogeneity (T80T20), and seed weight (WEIGHT). Clusterprofiler was used to perform a hypergeometric test using the Mapman functional terms. The *p*-values were converted to FDR *p*.adjust-values as shown in colors, the red color being more significant than the blue color. The dot size represents the gene ratio between the total gene number annotated in functional classes and the number of these genes present in your input list. GSEA, gene set enrichment analyses; GWAS, genome-wide association analyses; FDR, false discovery rate.

To pinpoint putative causal genes associated with significant QTNs, we identified from all surrounding SNPs located around QTNs, which showed high correlations due to linkage disequilibrium (LD) and could be linked to the phenotype. Using the PLINK algorithm (Purcell et al., 2007), we performed genome-wide correlations of significant QTNs (*P* < 10^−5^) with surrounding correlated SNPs with the threshold of 0.7 (*r*^2^ > 0.7) and located in a range of ±30 kb, corresponding to 2-fold the average LD decay in the HapMap population (Branca et al., 2011). As a result, we identified 120 putative causal genes related to the 73 QTNs for seed weight, 132 putative causal genes related to the 74 QTNs for germination speed (T50), and 109 putative causal genes related to the 63 QTNs for germination homogeneity (T80T20) (Supplementary Table 5). From these lists of candidate genes identified, we performed gene set enrichment analyses (GSEA) of functional classes to determine which processes could be involved in regulating the different seed traits (Figure 3). Interestingly, we identified significant enrichments of functional classes related to “HMG-CoA reductase” for germination speed; “secondary metabolism and chalcone synthase,” “subtilases,” “hormone metabolism of auxin and cytokinin” for germination homogeneity; and “RNA regulation of transcription” for seed weight.

To reduce these gene lists and refine the identification of putative causal genes, we combined these datasets with gene annotations from *Medicago* Genome Version 5 (Pecrix et al., 2018), transcriptomic data related to expression specificity in *M. truncatula* seeds (Chen et al., 2021a), and transcriptomic data during maturation of seeds developed both in optimal and heat stress conditions (Chen et al., 2021b; Supplementary Table 5). In consequence, we highlighted some candidate genes related to seed weight such as MtrunA17_Chr8g0392741, encoding a phosphatidylethanolamine-binding protein, homologous to *Arabidopsis MOTHER OF FT* (At1g18100, *MFT*), which showed a highly significant association with WEIGHT_C (*p* = 8.10^−20^) and WEIGHT_H (*p* = 4.10^−17^) and a strong differential expression during *M. truncatula* seed development between optimal and heat stress production (Supplementary Figure 3, indicated by red arrows and Supplementary Table 5). Another example of candidate gene regulated to seed weight during heat stress conditions was the *MtrunA17_Chr5g0403261* gene, closely related to *Arabidopsis DA1* gene (AT1G19270), known to regulate plant organ size, including seed size (Li et al., 2008), and also differentially expressed in the embryo between optimal and heat stress production. However, this gene did not display a single QTN with a high *p*-value but nine significant QTNs (*p* > 10^−5^). (Supplementary Figure 3, indicated by a green arrow).

In the subsequent part of this study, we decided to focus on candidate causal genes involved in germination speed/homogeneity. In chromosome 2 (Figure 2, indicated with black arrows), many QTNs associated with T50_H and T50_PL (>20 QTNs with *P* < 10^−5^) were found in the *MtrunA17_Chr2g0286331* gene, a member of a RING finger family containing a zinc-finger binding motif and ortholog of *Arabidopsis* of *MYB30-INTERACTING E3 LIGASE 1* (*MIEL1*, At5g18650). AtMIEL1 is a RING-type E3 ligase that plays a role in the proteasome pathway as a regulator of plant defense against bacteria (Marino et al., 2013) and ABA (Lee and Seo, 2016). In our study, *MtMIEL1* also showed a differential expression in endosperm between seeds produced under optimal and heat stress conditions, making a good candidate gene for further analyses. In chromosome 2 (Figure 2, indicated with red arrows), we identified another genomic interval displaying many QTNs identified in both T50_PL and T80T20_PL, which were more difficult to precisely relate to a specific gene sequence. These QTNs were spread on three closely located genes: MtrunA17_Chr2g0300271 encoding a nodule glycin-rich peptide, MtrunA17_Chr2g0300291 encoding a DEAD-box ATP-dependent RNA helicase, and *MtrunA17_Chr2g0300261* encoding a NF-YA3 transcription factor. However, our transcriptome data showed that only the *NF-YA3* transcription factor exhibited differential expression between seeds produced under optimal and heat stress conditions (Supplementary Table 5), which suggests that this potential pioneer gene could represent an interesting candidate regulator of plasticity of both germination speed and homogeneity. On chromosome 5 (Figure 2, indicated with a blue arrow), we also identified many QTNs associated with T80T20_PL and located in a genomic interval containing six closely located genes. Combination with our transcriptomic data allowed us to refine this list of putative causal genes to four candidates, as four of them displayed differential expression during seed development produced in optimal and heat stress conditions, but did not allow us to more precisely predict the causal gene.

### Functional Validation of *MIEL1* as a Regulator of Germination Plasticity of Seeds Produced Under Heat Stress

**Figure 4:**
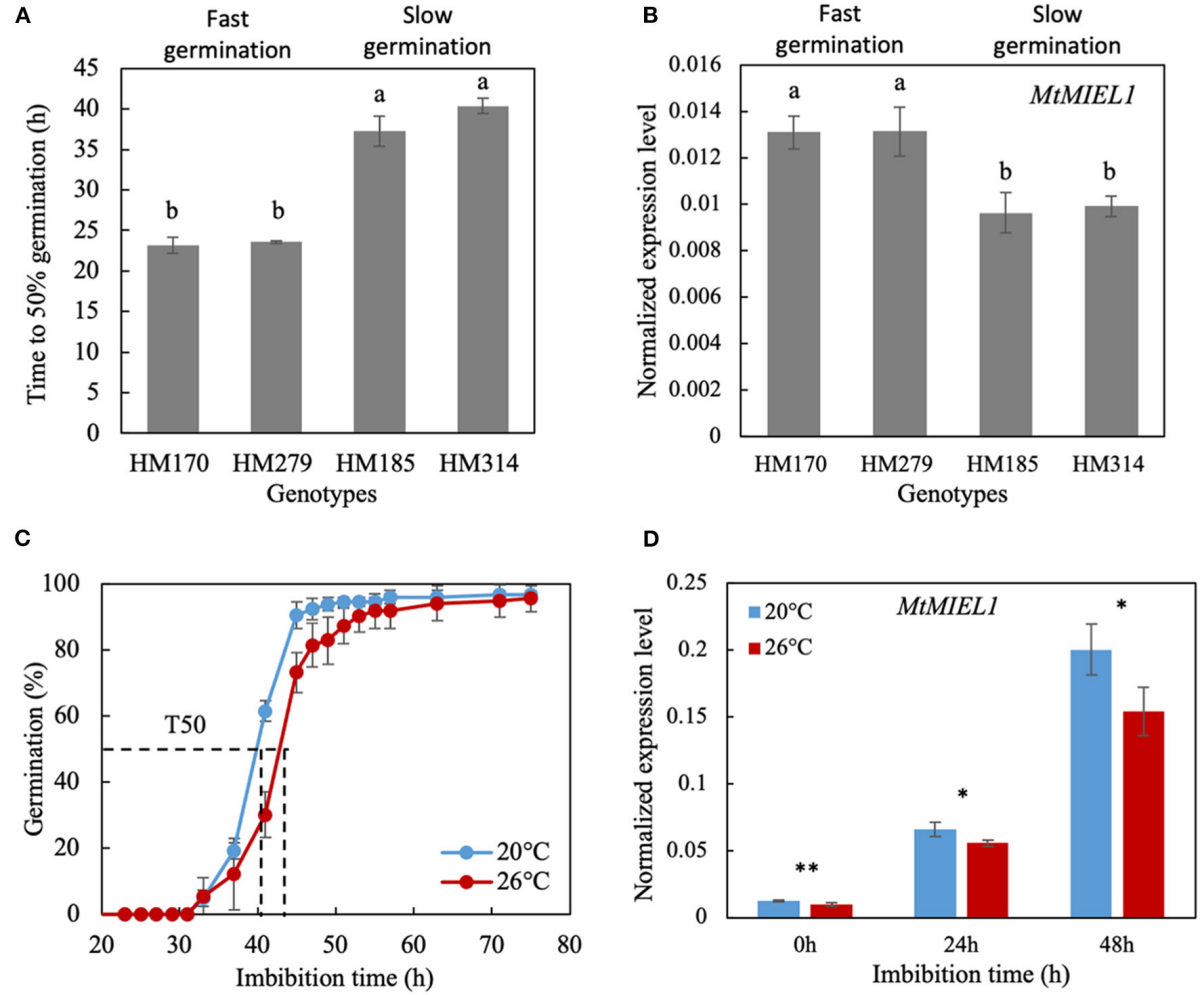
Characterization of seed germination and *MtMIEL1* expression in *Medicago truncatula* seeds. **(A)** Germination speed (T50) at 15°C of seeds produced under heat stress condition (26°C) of four natural *M. truncatula* HapMap accessions. **(B)** Expression level of *MtMIEL1* in dry, mature seeds produced under heat stress condition (26°C) of the four natural *M. truncatula* HapMap accessions. Different letters indicate significant differences (*P* < 0.05) identifying by ANOVA and Tukey's honest significant difference (HSD) test. **(C)** Germination curves at 10°C of *M. truncatula* reference genotype A17 seeds produced at 20°C and 26°C. The dash lines indicate the time to reach 50% germination (T50) of 20 and 26°C seeds. **(D)** Expression levels of *MtMIEL1* in dry, mature seeds (0 h), 24-h imbibed seeds (24 h), and 48-h imbibed seeds (48 h) which were produced at 20 and 26°C. *, 0.01 < *p*-value < 0.05; **, 0.001 < *p*-value < 0.01.

To further investigate the role of the candidate gene *MtMIEL1* (*MtrunA17_Chr2g0286331*) in the regulation of germination speed, we analyzed its expression profile in seeds of contrasting *M. truncatula* HapMap accessions showing slow and fast germination. First, we selected seeds HM170 and HM279 accessions as fast-germinating genotypes (i.e., T50 around 23 h) and HM185 and HM314 accessions as slow-germinating genotypes (i.e., T50 at 37 and 40 h, respectively; Figure 4A). Next, we extracted mRNA from their mature seeds produced under heat stress conditions. Fast-germinating genotypes displayed higher *MtMIEL1* relative transcript contents compared with slow-germinating genotypes (Figure 4B). Next, we assessed whether *MtMIEL1* would participate in the plasticity of germination speed in response to heat stress during seed production of the *M. truncatula* reference genotype A17. The germination speed of seeds produced at 26°C was significantly slower than seeds produced at 20°C (Figure 4C). Next, we performed transcript profiling of *MtMIEL1* at 0 h and 24 h of imbibition at 10°C in the dark (i.e., prior to radicle emergence). Figure 4D showed that at both times of imbibition, *MtMIEL1* transcripts were significantly higher in faster-germinating seeds than slower germinating seeds, consistent with the observations made on the four contrasting genotypes. From these results, we observed a positive correlation between *MtMIEL1* expression and speed of germination (i.e., higher *MtMIEL1* expression associated with faster germination) in five *M. truncatula* ecotypes following heat stress during seed production.

**Figure 5:**
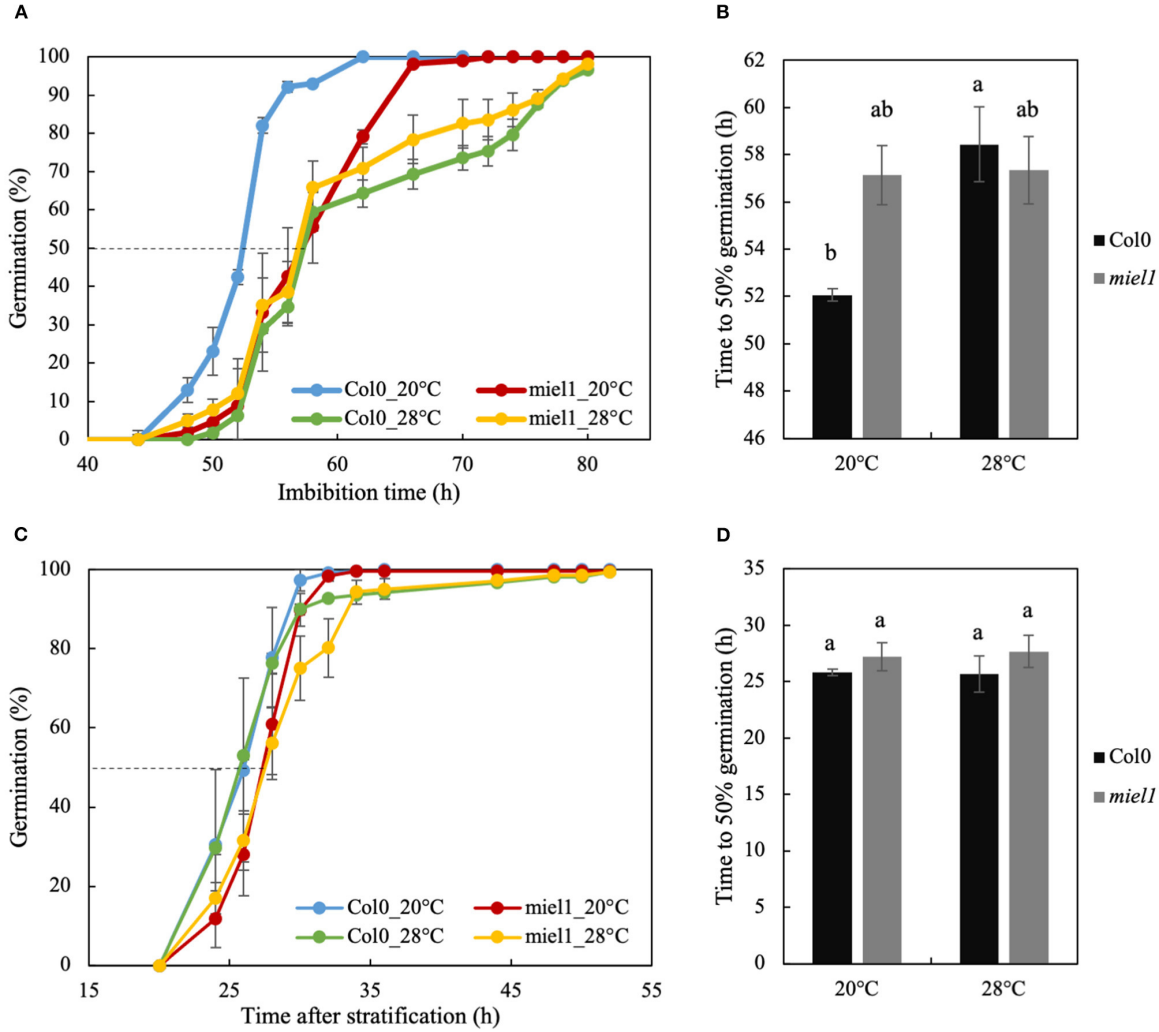
Seed germination of *Arabidopsis thaliana* wild-type (Col-0) and *miel1* T-DNA insertional mutant produced in optimal (20°C) and heat stress (28°C) conditions. **(A,B)** Germination curves and germination speed (T50) of freshly (dormant) harvested seeds of Col0 and *miel1* mutant grown in optimal (20°C) and heat stress (28°C) conditions. **(C,D)** Germination curves and germination speed (T50) of mature seeds from Col0 and *miel1* mutant after 72 h of stratification at 4°C to release dormancy. Error bars represent standard errors of the mean. Different letters in b and d indicate significant differences between samples (*P* < 0.05) identifying by ANOVA and Tukey's honestly significant difference test.

To validate the role of *MIEL1* in the regulation of germination speed and germination plasticity of seeds produced under heat stress conditions, we analyzed the ortholog of *MtMIEL1* in *Arabidopsis* by characterizing the germination kinetics from seeds produced at 20°C (control) and 28°C (heat stress) of the homozygote *miel1* mutants. The germination speed of freshly harvested wild-type seeds (Col0) produced at 28°C was much slower than that of seeds produced at 20°C (Figures 5A,B). In contrast, *miel1* mutant seeds germinated at the same speed regardless of the temperature experienced by the seeds during development, indicating that mutants had lost their plasticity. Moreover, we observed that the germination curves of *miel1* mutants were similar to that of wild-type seeds produced under heat stress (Figure 5A). Next, we repeated this experiment in non-dormant seeds obtained after a 72-h stratification treatment at 4°C to release dormancy. Wild-type and *miel1* mutant seeds displayed similar germination kinetics regardless of the production temperature (Figures 5C,D). These results obtained in *Arabidopsis* highlighted a new role of *MIEL1* as a regulator of germination speed in response to heat stress during seed development.

## Discussion

### Use of Natural Population and GWAS to Decipher Molecular Mechanisms Associated With Seed Traits

Natural variation within plant species causes phenotypic variations due to mutations generated by the evolutionary process. These natural variations are valuable resources to elucidate the molecular basis of phenotypic differences related to plant adaptation to distinct natural environments. In crops, phenotypic differences have been largely exploited in association genetic studies for QTL detection. Due to the development of sequencing technologies, many HapMap collections have been developed using the natural variations present in wild species, permitting genome-wide association studies to become a popular approach to correlate genotype to phenotype. Our study fully benefited from the *M. truncatula* HapMap collection with the help of post-GWAS and transcriptome analyses to understand how developing seeds cope with heat stress and modulate their germination response. This work extends previous studies showing that the temperature cues perceived by the mother plant are transmitted to their offspring (Penfield and MacGregor, 2017).

We characterized the genetic architecture that governs the plasticity response of *Medicago* and *Arabidopsis* seeds. We obtained a reasonable list of candidate genes potentially involved in regulating different seed traits. The GSEA from these candidate gene lists showed high relevance regarding the expected functional classes controlling the analyzed traits. For instance, candidate gene lists related to germination speed and germination homogeneity showed enrichment in genes functionally annotated as involved in “secondary metabolites–flavonoids–chalcone synthase,” “auxin and cytokinin hormone metabolisms,” and “subtilases.” The link between flavonoids and the plasticity response of germination is consistent with the sensitivity of the seed coat to temperature cues during development, which modulates the germination behavior (Penfield and MacGregor, 2017). It has been largely documented that chalcone synthase, the central enzyme of the flavonoid pathway, showing upregulation during the first 2–3 days of germination plays a role (Kubasek et al., 1992). Other studies confirmed the role of this pathway during germination using loss-of-function mutants of genes involved in flavonoid regulation, such as *TRANSPARENT TESTA GLABRA* (*TTG*), which displayed more efficient germination than wild-type seeds (Koornneef, 1981). Similar results were observed in different *TRANSPARENT TESTA* mutants (for review, Shirley, 1998). Second, the roles for auxin and cytokinin hormone metabolisms in germination performances were described in the literature. Indeed, even if auxin is not necessary for seed germination, it has been reported that IAA accumulated in the cotyledons of mature seeds (Epstein et al., 1986; Bialek and Cohen, 1989) influences seed germination with the interplay of ABA (Brady et al., 2003). This interplay was shown via miR160, which inhibits auxin-related gene expression during germination resulting in modulating ABA sensitivity during germination (Liu et al., 2007). Like auxin, cytokinin and cytokinin response factors play a role in enhancing seed germination when seeds were produced under stress (Khan and Ungar, 1997; Atici et al., 2005; Peleg and Blumwald, 2011).

Moreover, cytokinin was also demonstrated to play a role in the transition between dry seed and seedling in concert with ABA via *ABI5* gene regulation (Wang et al., 2011). Finally, enrichment of “subtilases” functional class in these candidate gene lists could be explained by the need of these proteases, which are highly active at very early stages of seed imbibition, regarding their role in the remobilization of storage proteins during seedling growth, as observed in barley (Galotta et al., 2019). It was not surprising to find enrichment of the “HMG-CoA reductase” class in the candidate gene list of germination speed. This central enzyme of the mevalonate and, therefore, isoprenoid pathway acts upstream to produce many important molecules such as secondary metabolites or hormones (e.g., ABA, GA, and cytokinin). However, despite its central and upstream position, a study reported an inhibitor of HMG reductase (i.e., one step even before the HMG-CoA reductase) retarded seed germination (Liao et al., 2014). In conclusion, by using the candidate gene lists obtained from the GWAS and GSEA, we could retrieve molecular mechanisms already described in the literature as directly or indirectly involved in studied seed traits, making GWA studies a reliable tool in exploratory analysis to decipher molecular processes controlling traits.

Furthermore, using GWAS and post-GWAS analyses combined with adequate transcriptomic data allowed us to identify solid candidate genes potentially regulating the different seed traits. For instance, an ortholog of the *Arabidopsis DA1* gene was identified as a candidate regulator of seed weight in *M. truncatula* (MtrunA17_Chr5g0403261, Supplementary Table 5). This gene, *DA1* (AT1G19270), has already been demonstrated to be a regulator of seed and organ size in *Arabidopsis* (Li et al., 2008). From this list of potentially reliable candidate genes, we also identified two of them strongly associated with seed germination performances, a *NUCLEAR FACTOR Y SUBUNIT A3* (*AtHAP2C/ NF-YA3, MtrunA17_Chr2g0300261*) and a RING-type zinc finger gene family (*MtrunA17_Chr2g0286331*), a potential ortholog of *Arabidopsis MIEL1* gene, that we called *MtMIEL1*.

### *MIEL1*, a Novel Regulator of Germination Plasticity of Seed Produced Under Heat Stress

The *MYB30-Interacting E3 Ligase1* (*MIEL1*) is an *Arabidopsis* RING-type E3 ubiquitin ligase identified to interact with and ubiquitinate MYB30, leading to MYB30 degradation via the proteasome pathway. It was first discovered as a regulator of plant defense response to bacteria as *MYB30* was known to trigger a hypersensitive response in the inoculated zone to restrict bacterial growth (Marino et al., 2013). More recently, it was shown to be involved in the protein turnover of another MYB protein, MYB96, a regulator of ABA signaling in seeds (Lee and Seo, 2016). It was reported that *miel1* mutants were hypersensitive to ABA compared with wild-type seeds, with *miel1* seeds that germinated 1.5-fold slower in the presence of 1 μM ABA compared with wild types (Lee and Seo, 2016). In contrast, without ABA treatment, they did not observe any difference in the germination of *miel1* mutants at 20°C. This result is similar to our observation using stratified (i.e., non-dormant) *miel1* seeds. Furthermore, we did not observe any significant change in germination (Figures 5C,D).

In contrast, we observed that in dormant seeds (i.e., with higher residual ABA content), *miel1* mutant seeds germinated significantly slower than wild-type seeds (Figure 5B), confirming the ABA hypersensitivity phenotype of the *miel1* seeds. In our study, we also observed a decrease of *MtMIEL1* expression in dry, mature seeds with the two *M. truncatula* HapMap accessions displaying slow germination compared with the two fast-germinating accessions (Figures 4A,B) and a lower *MtMIEL1* expression level during germination of *M. truncatula* A17 seeds produced under heat stress, which germinated slower, concerning seeds produced in optimal conditions (Figures 4C,D). Finally, in our study, we analyzed the impact of *miel1* mutation on germination kinetics of seeds produced under optimal and heat stress conditions. We found that *miel1* and wild-type seeds germinated at the same rate regardless of the environmental conditions of seed production (Figures 5A,B). Thus, our results strongly suggested that *MIEL1* plays a role in the germination plasticity of seeds produced under heat stress.

## Data Availability Statement

The original contributions presented in the study are included in the article/Supplementary Material, further inquiries can be directed to the corresponding author/s.

## Author Contributions

ZC, JL, BL, JB, and JV performed experiments. ZC, JB, OL, and JV analyzed data. ZC, OL, and JV wrote the manuscript. All authors reviewed the manuscript.

## Conflict of Interest

The authors declare that the research was conducted in the absence of any commercial or financial relationships that could be construed as a potential conflict of interest.
