# Supplementary material for: Genome-Wide Association Studies of Seed Performance Traits in Response to Heat Stress in Medicago truncatula Uncover MIEL1 as a Regulator of Seed Germination Plasticity
Source: Front Plant Sci. 2021 Jun 4;12:673072. doi: 10.3389/fpls.2021.673072 (PMC8213093; doi:10.3389/fpls.2021.673072)
Supplement: Supplementary file 1 [file Data_Sheet_1.zip › Supplementary Figure S1.pdf]

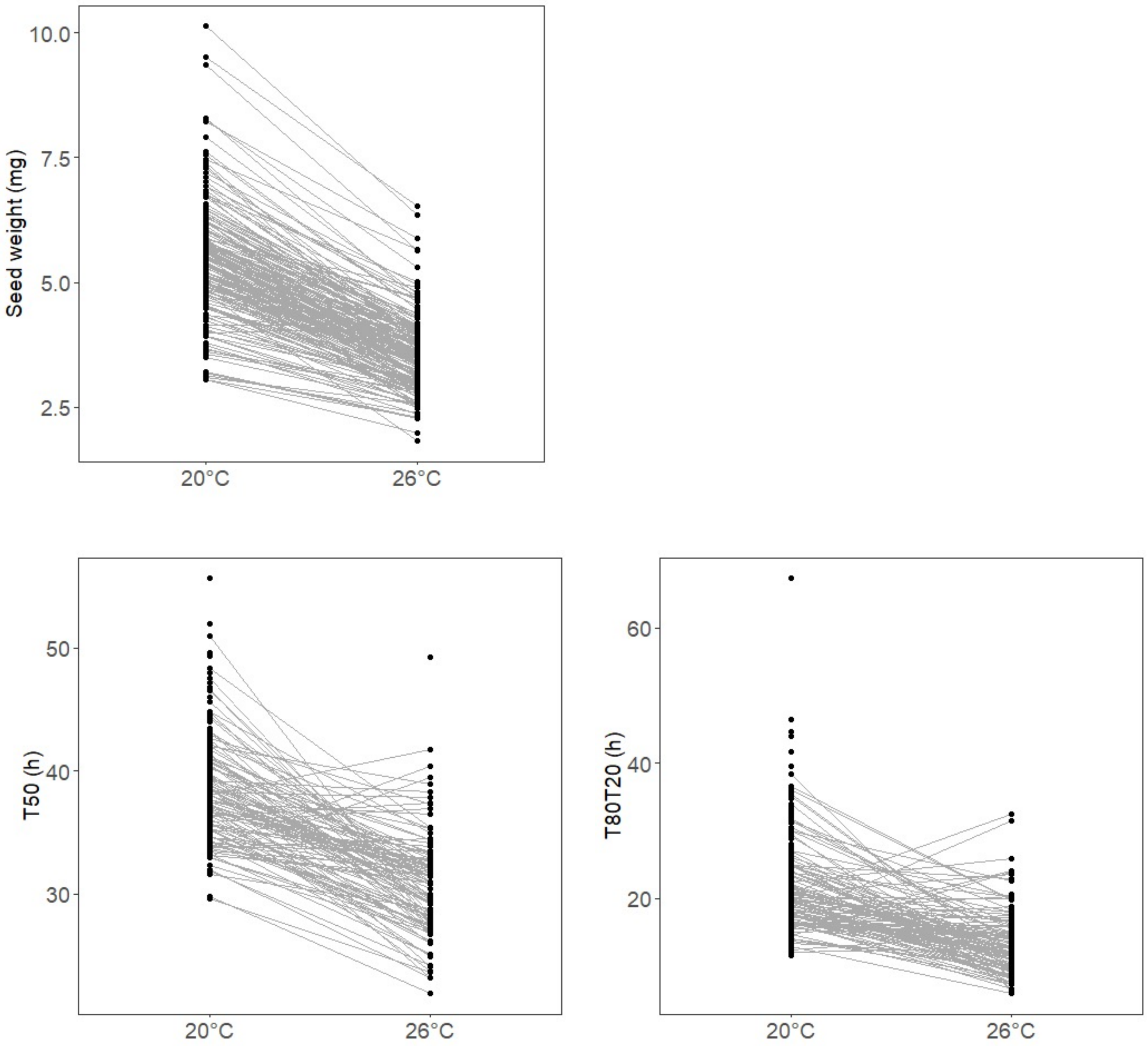

Figure S1: Graphical representations of phenotypic changes in seed traits of individual HapMap accessions between the two seed production conditions at 20°C (control) and 26°C (heat stress).
