# Supplementary material for: Genome-Wide Association Studies of Seed Performance Traits in Response to Heat Stress in Medicago truncatula Uncover MIEL1 as a Regulator of Seed Germination Plasticity
Source: Front Plant Sci. 2021 Jun 4;12:673072. doi: 10.3389/fpls.2021.673072 (PMC8213093; doi:10.3389/fpls.2021.673072)
Supplement: Supplementary file 1 [file Data_Sheet_1.zip › Supplementary Figure S2.pdf]

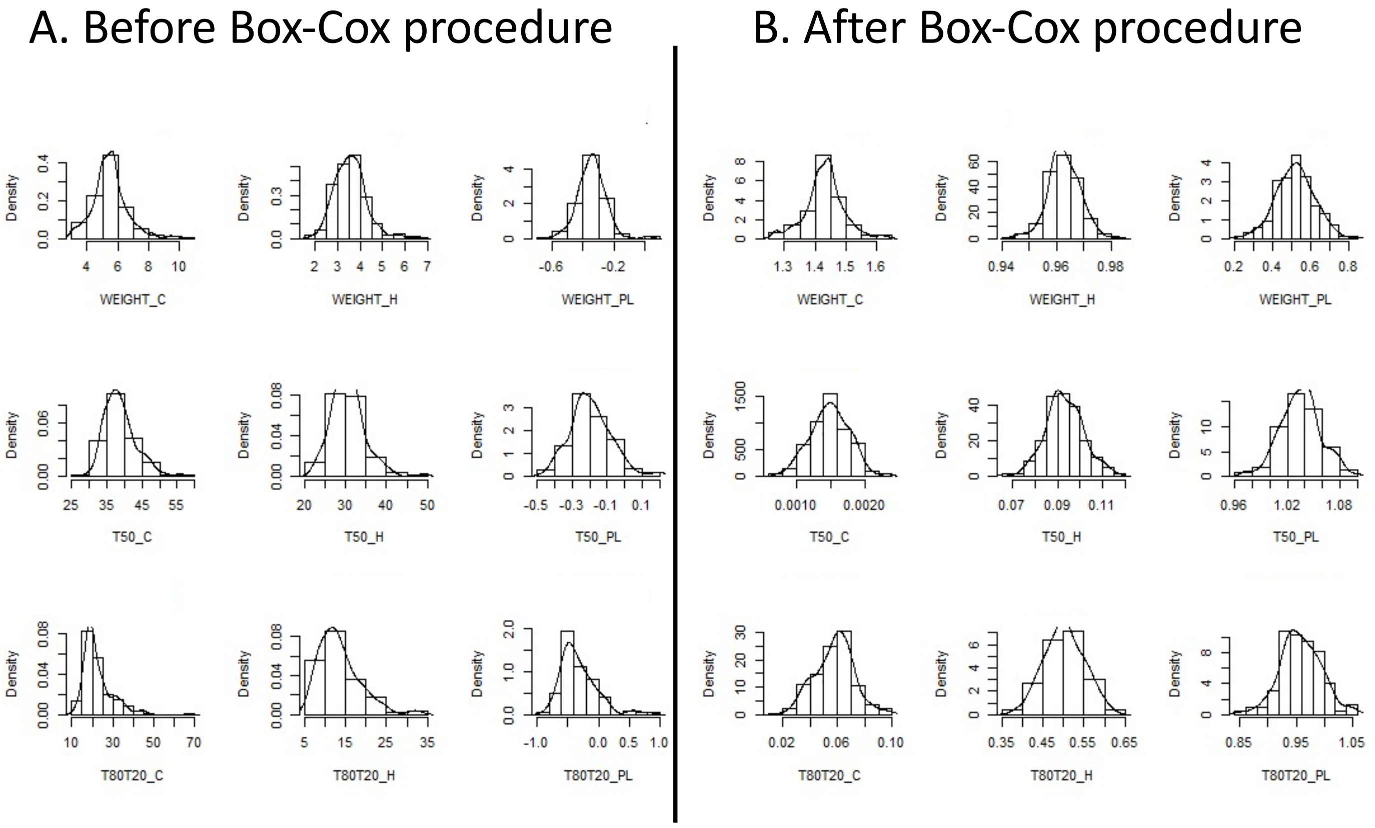

Figure S2: Distribution histograms before (A) and after (B) the Box-Cox procedure to normalize phenotypic data of seed traits. Corresponding distribution curves are indicated on histograms. Traits are indicated on the x-axis and correspond to seed weight (WEIGHT), germination speed (T50) and germination homogeneity (T80T20) with _C (20°C) for control conditions, _H for heat stress (26°C) conditions and _PL for plasticity.
